# Pediatric erythromelalgia from multidisciplinary perspectives: a scoping review

**DOI:** 10.1038/s41390-025-03817-4

**Published:** 2025-01-16

**Authors:** Don Daniel Ocay, Maria Graziano Maloney, Genevieve D’Souza, Catherine A. Brownstein, Jacqui Clinch, Dawn Marie Davis, Deirdre De Ranieri, Carolina Donado, Meghan Halpin, Deepa Kattail, Benjamin Howard Lee, Kimberly Lobo, Danielle Ravetti, Paola Sandroni, Jennifer N. Stinson, See Wan Tham, Gary A. Walco, Suellen M. Walker, Timothy W. Yu, Charles B. Berde

**Affiliations:** 1 Department of Anesthesiology, Critical Care and Pain Medicine, Boston Children’s Hospital, Boston, MA USA; 2 Department of Anaesthesia, Harvard Medical School, Boston, MA USA; 3 F.M. Kirby Neurobiology Center, Boston Children’s Hospital, Boston, MA USA; 4 Department of Anesthesiology, Perioperative and Pain Medicine, Stanford University, Stanford, CA USA; 5 Division of Genetics and Genomics, Department of Pediatrics, Boston Children’s Hospital, Boston, MA USA; 6 Department of Pediatrics, Harvard Medical School, Boston, MA USA; 7 Department of Pediatric Rheumatology, Bristol Royal Hospital for Children, Bristol, United Kingdom; 8 Department of Dermatology, Mayo Clinic Rochester, Rochester, MN USA; 9 Department of Pediatric and Adolescent Medicine, Mayo Clinic Rochester, Rochester, MN USA; 10 Department of Pediatrics, Northwestern University Feinberg School of Medicine, Chicago, IL USA; 11 Department of Anesthesia and Pain Medicine, The Hospital for Sick Children (Sickkids), Toronto, ON Canada; 12 Department of Anesthesiology, Emory University School of Medicine, Atlanta, GA USA; 13 Patient Partner, Lodi, CA USA; 14 The Erythromelalgia Association, Lodi, CA USA; 15 Department of Neurology, Mayo Clinic Rochester, Rochester, MN USA; 16 Research Institute, The Hospital for Sick Children (SickKids), Toronto, ON Canada; 17 Department of Anesthesiology and Pain Medicine, University of Washington School of Medicine, Seattle, WA USA; 18 Department of Anesthesiology and Pain Medicine, Seattle Children’s Hospital, Seattle, WA USA; 19 Developmental Neurosciences, University College London Great Ormond Street Institute of Child Health, London, United Kingdom; 20 Department of Paediatric Anaesthesia and Pain Medicine, Great Ormond Street Hospital NHS Foundation Trust, London, United Kingdom

## Abstract

**Abstract:**

Erythromelalgia is a rare, chronic pain disorder characterized by the triad of intense burning sensation, warmth, and redness, primarily involving the hands and feet, and usually alleviated by cold and worsened by heat. The objective of this scoping review was to: 1) map the existing literature on erythromelalgia in youth, 2) identify knowledge gaps, and 3) inform directions for future research in pediatric erythromelalgia. One hundred and sixty-seven studies reporting 411 cases of childhood-onset erythromelalgia were identified. Variability was found in reporting of clinical symptoms, the clinical presentations and diagnostic criteria used for classification of erythromelagia, the clinical assessments and investigations performed, and the types of interventions and management plans utilised. While factors to aid early recognition and optimize management have been identified, there are also significant gaps for future research to address. Ongoing efforts to develop a multicenter registry of pediatric erythromelalgia cases, with standardized data collection and reporting, will be beneficial to establish consensus recommendations for the diagnosis and management of pediatric erythromelalgia.

**Impact:**

- This scoping review maps the existing literature on pediatric erythromelalgia.
- Variability was found in reporting of clinical symptoms, the clinical presentations and diagnostic criteria used for classification of erythromelagia, the clinical assessments and investigations performed, and the types of interventions and management plans utilised.
- The development of an international registry would immensely benefit multidisciplinary experts involved in the care of pediatric erythromelalgia and those with lived experience.

## Introduction

Erythromelalgia was first described in a 1878 paper by Silas Weir Mitchell entitled, “On a rare vasomotor neurosis of the extremities and on the maladies with which it may be confounded.“^1^ The term “erythromelalgia” was derived from the Greek words: “erythros” = red, “melos” = extremity, and “algos” = pain. In Mitchell’s observations, patient symptoms were primarily in the feet and characterized by a burning sensation that was alleviated by cold and exacerbated by warmth or physical activity, and associated with redness.

Since then, the diagnostic criteria for erythromelalgia have evolved. In 1932, Brown classified some cases as secondary to other underlying diseases and others as “primary”.^2^ He proposed four fundamental criteria for the diagnosis of erythromelalgia: “1) bilateral burning pain in the extremities, 2) sharp increase of local heat in the affected parts, but redness, flushing or congestion may vary in degree, 3) production and aggravation of the distress by heat and exercise, and 4) relief by rest, cold and elevation.”^2^ In 1938, Smith and Allen proposed substituting the term “erythromelalgia” for “erythermalgia” denoting the importance of the hot burning sensations.^3^ In 1994, Drenth and Michiels proposed three classifications: 1) erythromelalgia in thrombocythemia, 2) primary erythermalgia, and 3) secondary erythermalgia.^4^ More recently, the recognition that some cases of familial erythromelalgia are linked to dominant gain-of-function mutations of the *SCN9A* gene led to the introduction of the term “inherited erythromelalgia” referring to patients with a confirmed genetic variant, leaving “symptomatic erythromelalgia” as a descriptor for cases without a confirmed genetic variant.^5^

Today, erythromelalgia and erythermalgia are used interchangeably to describe a rare, chronic pain disorder characterized by the triad of intense burning sensation, warmth, and erythema, primarily involving the distal extremities (hands and feet), and usually alleviated by cold and worsened by heat.^6,7^ Patients with these findings commonly experience prolonged delay in diagnosis, suffer from missed diagnosis or remained undiagnosed. They are referred and evaluated across multiple subspecialties such as pain medicine, neurology, rheumatology, dermatology, and genetics.^8^ With an estimated incidence of 0.25-2 cases per 100,000 per year,^9,10^ it is a rare condition that is also associated with high morbidity. The rarity of the condition has led to few case series or cohort studies on pediatric erythromelalgia, and even fewer on their longitudinal trajectories.^5,8,11^ Overcoming these knowledge gaps in the etiology of erythromelalgia offers potential to develop targeted therapies to reduce pain and associated co-morbidities.

A comprehensive review encompassing all potential etiologies or presentations of pediatric erythromelalgia has not been published. General reviews have been written, but did not focus on clinical presentations in children.^12,13^ Therefore, the objective of this scoping review was to: 1) map the existing literature on erythromelalgia in children and adolescents, 2) identify knowledge gaps, and 3) inform directions for future research in pediatric erythromelalgia.

## Methods

The scoping review was conducted following the Preferred Reporting Items for Systematic Reviews and Meta-Analyses extension for Scoping Reviews,^14^ and pre-registered on Open Science Framework.^15^

We searched five online databases: PubMed, Embase, Web of Science, CINAHL, and Cochrane. The selection of articles was made through the following search string: (“erythromelalgia” OR “erythermalgia”). Articles published from inception until November 30, 2023 were extracted on December 11, 2023.

Two reviewers independently screened the titles and abstracts of all extracted articles matching the research aim and inclusion criteria, excluding duplicates, reviews, commentaries, posters, and proceeding papers, using Covidence (www.covidence.org). Original English pediatric (< 18 years old), single case studies, case series (i.e. ≥ 2 cases presented), and cohort studies, or adult single case studies, case series, and cohort studies with symptoms that emerged at the age of 18 years old or younger were identified for full-text screening. The reference list of all screened reviews and full-text articles were checked for additional papers matching the inclusion criteria. References deemed relevant by at least one reviewer underwent full-text screening. Any disagreements were resolved by a third independent reviewer.

Study characteristics (e.g. year of publication, country of authors, type of study) and all available data on demographics, clinical features, genetic and laboratory testing results, comorbidities, and management were extracted, entered, and cross-checked in a database on Covidence. Unreported data were labeled “not reported”. All data are presented as the frequency of studies (S) or cases (C), unless otherwise specified.

## Results

### Study selection

A total of 3,081 references were identified through database searching (Supplementary Fig. 1A**)**. After removal of duplicates, 1,848 titles or abstracts were screened, leading to 732 articles that underwent full-text review. Eighteen additional articles, identified through the reference lists of screened reviews and articles, underwent full-text review, and were assessed for eligibility. There were 193 articles retained for data extraction. Pediatric-specific data could not be extracted in 19 articles, but their overall findings are summarized in Supplementary Table 1. Overlap in case reports led to 167 studies retained for analysis (Supplementary Table 2).

### Study characteristics

The studies identified were primarily conducted in North America (S = 59), Europe (S = 53), and Asia (S = 34), followed by South America (S = 4), and Australia (S = 4). There were 11 multi-national studies as follows: United States and China (S = 4), Japan (S = 2) or Taiwan (S = 1), Netherlands and Belgium (S = 1) or Canada (S = 1), United Kingdom, United States and Germany (S = 1), and Germany, Norway and Sweden (S = 1). The affiliation of the authors of one study was not reported. The types of studies published included case reports (S = 100), case series (S = 41), translational studies (S = 22), case-control studies (S = 2), and randomized, double-blind, placebo-controlled, crossover trials (S = 2). Although the first confirmed case of pediatric erythromelalgia was published in 1878, most of the studies identified (S = 125) reporting pediatric cases of erythromelalgia were published in the last two decades (Supplementary Fig. 1B).

Childhood-onset erythromelalgia were met for 411 cases reported in these studies, in which 148 were male, 229 were female, and 34 were not reported. Race and ethnicity were not reported for most cases (C = 355), but the race of those reported included Asian (n = 19), Black or African American (n = 1), Hispanic or Latino (n = 1), and White (n = 35). Familial inherited erythromelalgia was reported in 51 studies, while spontaneous or de novo erythromelalgia was reported in 48 studies. Family history was not reported in 68 studies. Overall, the studies comprise 189 cases with primary inherited erythromelalgia (confirmed family history or genetic mutation), 194 with primary symptomatic erythromelalgia, and 28 with secondary erythromelalgia (6 Olmsted syndrome, 7 small fiber neuropathy, 3 small fiber neuropathy and Familial Mediterranean fever, 1 small fiber neuropathy and Behcet’s disease, 6 acute secondary erythromelalgia, 1 red ear syndrome – erythromelalgia type, 2 erythrocyanosis, 1 painful redness of feet due to chilblains, and 1 DiGeorge and CHARGE syndromes).

### Outcomes reported

#### Clinical presentation

Erythromelalgia was usually diagnosed based on clinical history and physical examination. The diagnostic approach was primarily focused on excluding other underlying diseases, and some studies also included genetic testing. Overall, the main criteria for diagnosis of erythromelalgia followed Brown’s 1932 criteria which includes: 1) burning pain of the extremities, 2) pain aggravated by warming and exercise, 3) pain relieved by cooling, rest or elevation, 4) redness of the affected skin, and 5) increased temperature of the affected skin. However, only 53 studies reported all five diagnostic criteria in their cases (Table 1) representing 163 of all cases reported. Relevant physical examination findings were also documented for many patients: swelling/edema was reported in 69 studies (C = 108), and skin injury (ulcers, lesions, blisters, maceration, erosion, and cracking) was reported in 59 studies (C = 152).

**Table 1:** Characteristics of cases reported in studies.

	Number of studies (S = 167)	Number of cases (C = 411)
**Diagnostic criteria**		
“Burning” pain	128 (76.6)	293 (71.3)
Pain aggravated by warming and exercise	111 (66.5)	245 (59.6)
Pain relieved by cooling, rest or elevation	139 (83.2)	289 (70.3)
Redness of the affected skin	154 (92.2)	364 (88.6)
Increased temperature of the affected skin	100 (59.9)	208 (50.6)
**Affected body location**		
Face	17 (10.2)	46 (11.2)
Ears	22 (13.2)	45 (10.9)
Hands	100 (59.9)	236 (57.4)
Hands with arms	15 (9.0)	27 (6.6)
Feet	151 (90.4)	358 (87.1)
Feet with legs	63 (37.7)	117 (28.5)
Both hands and feet	97 (58.1)	230 (56)
Other locations	10 (6.0)	10 (2.4)
**Comorbidities**		
Inflammatory	16 (9.6)	18 (4.4)
Acne	3 (1.8)	3 (0.7)
Arthritis	1 (0.6)	1 (0.2)
Calcaneal apophysitis	1 (0.6)	1 (0.2)
Celiac disease	2 (1.2)	2 (0.5)
Cellulitis	2 (1.2)	2 (0.5)
Dermatitis	2 (1.2)	2 (0.5)
Enlarged lymph nodes	1 (0.6)	1 (0.2)
Esophagitis,	1 (0.6)	1 (0.2)
Familial Mediterranean fever	1 (0.6)	1 (0.2)
Hashimoto’s disease	1 (0.6)	1 (0.2)
Interstitial cystitis	1 (0.6)	1 (0.2)
Peptic ulcer	1 (0.6)	1 (0.2)
Systemic lupus erythematosus	1 (0.6)	1 (0.2)
Tonsillitis	1 (0.6)	1 (0.2)
Urticaria	1 (0.6)	1 (0.2)
Neurologic	12 (7.2)	17 (4.1)
Autonomic dysfunction	1 (0.6)	4 (1.0)
Developmental delay	6 (3.6)	7 (1.7)
Fainting	2 (1.2)	2 (0.5)
Hyperhydrosis	1 (0.6)	1 (0.2)
Mute	1 (0.6)	1 (0.2)
Restless leg syndrome	2 (1.2)	2 (0.5)
Seizures	3 (1.8)	3 (0.7)
Spina bifida	2 (1.2)	2 (0.5)
Vascular	10 (6.0)	27 (6.6)
Anemia	1 (0.6)	2 (0.5)
Cardiac disease	1 (0.6)	1 (0.2)
Chilblains	1 (0.6)	1 (0.2)
Erythrocyanosis	1 (0.6)	2 (0.5)
Hypertension	8 (4.8)	18 (4.4)
Hypokalemia	1 (0.6)	1 (0.2)
Phenylketonuria	1 (0.6)	1 (0.2)
Popliteal artery entrapment syndrome	1 (0.6)	1 (0.2)
Other	25 (15.0)	40 (9.7)
Amenorrhea	1 (0.6)	1 (0.2)
Asthma	1 (0.6)	1 (0.2)
Chronic cough and nasal obstruction	1 (0.6)	1 (0.2)
Chronic kidney disease.	1 (0.6)	1 (0.2)
Chronic respiratory failure	1 (0.6)	1 (0.2)
Constipation	1 (0.6)	1 (0.2)
Diabetes mellitus	3 (1.8)	5 (1.2)
Diabetic ketoacidosis	1 (0.6)	1 (0.2)
Diarrhea	3 (1.8)	3 (0.7)
DiGeorge and CHARGE syndromes	1 (0.6)	1 (0.2)
Dysphagia	1 (0.6)	1 (0.2)
Endometriosis	1 (0.6)	1 (0.2)
Exophthalmos	1 (0.6)	1 (0.2)
Fibromyalgia / Myalgia	2 (1.2)	3 (0.7)
Gastroesophageal reflux disease	1 (0.6)	1 (0.2)
Glaucoma	1 (0.6)	1 (0.2)
Headaches	2 (1.2)	3 (0.7)
Hexadactylism	1 (0.6)	1 (0.2)
Hyperlipidemia	1 (0.6)	2 (0.5)
Hypermobility	1 (0.6)	1 (0.2)
Hyporeflexia	1 (0.6)	1 (0.2)
Hypothyroidism	3 (1.8)	5 (1.2)
Hypotonia,	2 (1.2)	2 (0.5)
Ichtyosis	1 (0.6)	1 (0.2)
Joint hyperextensibility	1 (0.6)	1 (0.2)
Megalocornea	1 (0.6)	1 (0.2)
Micrognathia	1 (0.6)	1 (0.2)
Musculoskeletal pain	1 (0.6)	1 (0.2)
Olmsted syndrome	1 (0.6)	2 (0.5)
Overweight / Obesity	2 (1.2)	2 (0.5)
Pituitary adenoma	1 (0.6)	1 (0.2)
Trichotillomania	1 (0.6)	1 (0.2)
Vesico-uretero-renal reflux disease	1 (0.6)	1 (0.2)
**Psychosocial factors**		
Not reported	149 (89.2)	
Reported	18 (10.8)	65 (15.8)
Anxiety	6 (3.6)	17 (4.1)
Behavioral problems (e.g. Obsessive-compulsive behavior, Oppositional defiant disorder, Attention deficit disorder with hyperactivity)	9 (5.4)	11 (2.7)
Depression	8 (4.8)	17 (4.1)
Emotional distress	3 (1.8)	5 (1.2)
Fatigue	2 (1.2)	2 (0.5)
Pain catastrophizing	2 (1.2)	6 (1.5)
Suicidal ideation	6 (3.6)	6 (1.5)
**Impaired quality of life**		
Not reported	60 (35.9)	
Reported	107 (64.1)	211 (51.3)
Difficulty sleeping	29 (17.4)	42 (10.2)
Limited physical activity	91 (54.5)	186 (45.3)
Limited social activity	17 (10.2)	31 (7.5)
Lifestyle change	23 (13.8)	31 (7.5)
Prefer to walk barefoot	21 (12.6)	30 (7.3)

Data presented as *n* (%).

The studies reported cases mainly affected in their hands (C = 236), feet (C = 358), and ears (C = 45), or hands and feet (C = 230) (Table 1). Other locations included the neck, trunk and groin. Most of the studies did not report any inciting event (S = 130) or reported the spontaneous onset of erythromelalgia symptoms (C = 10). Reported inciting events (C = 62) prior to the erythromelalgia symptoms included illness (C = 27), infection (C = 33), physical activity (C = 23), trauma (C = 6), surgery (C = 1), post-vaccination (C = 2), ingestion of *Agaricus spp. and Lepista inversa (Scop.) Pat* mushrooms (C = 1), and cessation of norephedrine therapy (C = 1). The medical history was not reported in 276 cases and noted as unremarkable in 55 cases. The remaining 80 cases with reported comorbidities (Table 1) included inflammatory (C = 18), neurologic (C = 17), vascular (C = 27), or other conditions (C = 40).

Psychosocial factors were assessed in 23 studies, but findings were reported in only 21 studies, which primarily included anxiety, behavioral problems, depression, and suicidal ideation (Table 1). Poor school/work attendance or performance was reported in 31 studies (C = 65). Impaired quality of life was reported in 107 studies (C = 211), which included difficulty sleeping, limited physical activity, limited social activity, lifestyle change (e.g. wheelchair-bound, loss of autonomy, moving environment), and preference to walk barefoot (Table 1).

#### Evaluation of genetic mutations

From this search, the first pediatric case of inherited erythromelalgia linked to a dominant gain-of-function mutation of the *SCN9A* gene was reported in 2004.^16^ Since then, only 70 studies have reported genetic testing, with 58 studies reporting a genetic candidate linked to the clinical profile of the cases. In 144 cases with childhood-onset erythromelalgia, 32 *SCN9A* gene mutation variants were reported, while 9 non-*SCN9A* genetic mutations were reported for 11 cases (Table 2).

**Table 2:** Genetic mutations reported in pediatric cases.

	Number of studies (S = 58)	Number of cases (C = 155)	Inheritance		
			Confirmed mutation in family	De novo	Not reported
**Non-*SCN9A*** **gene mutations**	5 (8.6)	11 (7.1)			
*MEFV*^a^	1 (1.7)	3 (1.9)			
A457V	1 (1.7)	1 (0.6)	1 (100)	0	0
E148Q	1 (1.7)	2 (1.3)	1 (50.0)	0	1 (50.0)
M694V	1 (1.7)	1 (0.6)	1 (100)	0	0
P369S	1 (1.7)	1 (0.6)	1 (100)	0	0
R408Q	1 (1.7)	1 (0.6)	1 (100)	0	0
*NMNAT2*	1 (1.7)	2 (1.3)			
T94M	1 (1.7)	2 (1.3)	2 (100)	0	0
*TRPV3*	3 (5.2)	6 (3.9)			
G568C	2 (3.4)	4 (2.6)	4 (100)	0	0
L673F	2 (3.4)	2 (1.3)	0	2 (100)	0
Q216_G262del	1 (1.7)	2 (1.3)	2 (100)	0	0
***SCN9A*** **gene mutations**	53 (91.4)	144 (92.9)			
A1632G	1 (1.7)	1 (0.6)	1 (100)	0	0
A1632T	2 (3.4)	2 (1.3)	2 (100)	0	0
A1746G	1 (1.7)	1 (0.6)	0	1 (100)	0
A863P	1 (1.7)	1 (0.6)	0	1 (100)	0
F1449V	5 (8.6)	26 (16.8)	24 (92.3)	0	2 (7.7)
F216S	4 (6.9)	4 (2.6)	1 (1.3)	1 (1.3)	2 (50.0)
F826Y	2 (3.4)	4 (2.6)	4 (100)	0	0
G236W	1 (1.7)	1 (0.6)	0	1 (100)	0
G856D	1 (1.7)	1 (0.6)	0	0	1 (100)
G856R	1 (1.7)	2 (1.3)	2 (100)	0	0
I136V	5 (8.6)	9 (5.8)	2 (22.2)	0	7 (77.8)
I234T	3 (5.2)	3 (1.9)	0	3 (100)	0
I848T	10 (17.2)	11 (7.1)	3 (27.3)	3 (27.3)	5 (45.5)
L245V	2 (3.4)	10 (6.5)	4 (40.0)	0	6 (60.0)
L823R	1 (1.7)	1 (0.6)	0	0	1 (100)
L858F	7 (12.1)	11 (7.1)	6 (54.5)	1 (9.1)	4 (36.4)
L858H	2 (3.4)	2 (1.3)	2 (100)	0	0
L869F	1 (1.7)	1 (0.6)	0	1 (100)	0
L951I	1 (1.7)	1 (0.6)	0	1 (100)	0
L955del	1 (1.7)	1 (0.6)	1 (100)	0	0
N395K	2 (3.4)	3 (1.9)	3 (100)	0	0
P1308L	2 (3.4)	2 (1.3)	0	0	2 (100)
P610T^b^	1 (1.7)	1 (0.6)	1 (100)	0	0
Q10R	1 (1.7)	1 (0.6)	0	1 (100)	0
Q875E	3 (5.2)	3 (1.9)	0	2 (66.7)	1 (33.3)
R1150W	1 (1.7)	1 (0.6)	1 (100)	0	0
R220P	1 (1.7)	7 (4.5)	2 (28.6)	0	5 (71.4)
S241T	4 (6.9)	10 (6.5)	9 (90.0)	0	1 (10.0)
S449N^c^	1 (1.7)	1 (0.6)	0	1 (100)	0
V1316A	6 (10.3)	6 (3.9)	1 (16.7)	2 (33.3)	3 (50.0)
V400M	4 (6.9)	11 (7.1)	8 (72.7)	1 (9.1)	2 (18.2)
V872G	1 (1.7)	1 (0.6)	0	0	1 (100)
Unknown variant	3 (5.2)	6 (3.9)	0	0	6 (100)

Data presented as *n* (%).

*SCN9A* Sodium voltage-gated channel alpha subunit 9, *MEFV* Mediterranean fever, *NMNAT2* Nicotinamide nucleotide adenylyltransferase 2, *TRPV3* Transient receptor potential vanilloid-3.

^a^One case had the A457V, E148Q, P369S, and R408Q variants.

^b^P610T variant was present in a case with the L858F variant.

^c^S449N variant was present in a case with the I848T variant.

#### Clinical investigations

Laboratory tests were reported in 106 studies (Table 3). Abnormal laboratory findings included: high platelet count, increased erythrocyte sedimentation rate and/or C-reactive protein, positive antinuclear antibodies, high white blood cells, defect in immunity (e.g. low levels of immunoglobulins or complement deficiency), and elevated liver enzymes (Supplementary Table 3). Neurological examinations were reported in 110 studies (Table 3). Significant findings included: abnormal quantitative sensory testing results, sudomotor dysfunction, abnormal nerve conduction, small fiber neuropathy, hypertension (i.e. high resting blood pressure), and low intraepidermal nerve fiber density in skin biopsies (Supplementary Table 3). Other significant non-neurological findings from skin biopsies included inflammation of the blood vessels (e.g. leukocytoclastic vasculitis, strong phospho-extracellular signal-regulated kinase expression, perivascular or interstitial infiltrate, thick capillary walls) and hyperkeratosis. Vascular studies were reported in 55 studies (Table 3). Abnormal vascular findings included: increased blood flow and associated increased temperature, decreased blood flow, and abnormal morphology (Supplementary Table 3). Imaging studies were reported in 52 studies (Table 3). Abnormal imaging findings included: bone loss (C = 2), scans suggestive of reflex sympathetic dystrophy or complex regional pain syndrome (C = 2, e.g. bone marrow edema and increased tracer accumulation in the affected areas at the vascular, blood pool and bone phases), spina bifida occulta (C = 1), structural or functional changes in the head/neck at rest (C = 5, e.g. focal epileptiform discharges with diffuse background slowing, cervical spondylopathy, asymmetry in the anterior horns of the ventricles, abnormalities of the pituitary stalk, a small adenohypophysis, reduced depth of the sella turcica, or cerebral infarctions) or during treatment/pain relief (C = 2, e.g. changes in cerebral blood flow between baseline pain and cooling relief, or shift in brain activity during carbamazepine treatment from valuation (i.e. decision-making) and pain areas toward primary somatosensory-motor and parietal attention areas).

**Table 3:** Clinical investigations reported in studies.

	Number of studies with reported results (S = 167)	Number of cases with reported results (C = 411)	Case findings (C = 411)		
			Normal	Abnormal	Not reported
**Laboratory tests**	106 (63.5)				
Complete blood count	72 (43.1)	146 (35.5)	113 (77.4)	32 (21.9)	1 (0.7)
C-reactive protein	21 (12.6)	22 (5.4)	17 (77.3)	5 (22.7)	0
Erythrocyte sedimentation rate	36 (21.6)	86 (20.9)	74 (86.0)	12 (14.0)	0
Immune / autoimmune tests^a^	57 (34.1)	82 (20.0)	65 (79.3)	16 (19.5)	1 (1.2)
Iron and total iron-binding capacity	1 (0.6)	1 (0.2)	0	1 (100)	0 (0)
Prothrombin time / International normalized ratio	8 (4.8)	8 (1.9)	7 (87.5)	0	1 (12.5)
Rheumatoid factor	20 (12.0)	32 (7.8)	31 (96.9)	1 (3.1)	0
Vitamin B12	8 (4.8)	24 (5.8)	21 (87.5)	3 (12.5)	0
Vitamin D	2 (1.2)	20 (4.9)	7 (35.0)	13 (65.0)	0
Lipid panel	12 (7.2)	12 (2.9)	9 (75.0)	2 (16.7)	1 (8.3)
Metabolic panel	45 (26.9)				
Electrolytes	14 (8.4)	14 (3.4)	12 (85.7)	1 (7.1)	1 (7.1)
Glucose	16 (9.6)	17 (4.1)	16 (94.1)	1 (5.9)	0
Renal function tests	24 (14.4)	25 (6.1)	20 (80.0)	4 (16.0)	1 (4.0)
Hepatic function tests	28 (16.8)	31 (7.5)	25 (80.6)	5 (16.1)	1 (3.2)
Proteins	14 (8.4)	45 (10.9)	25 (55.6)	1 (2.2)	19 (42.2)
Unspecified	11 (6.6)	13 (3.2)	13 (100)	0	0
Thyroid function tests	13 (7.8)	33 (8)	30 (90.9)	3 (9.1)	0
Urinalysis	28 (16.8)	32 (7.8)	26 (81.3)	6 (18.8)	0
Other	46 (27.5)	71 (17.3)	59 (83.1)	11 (15.5)	1 (1.4)
Unspecified*	14 (8.4)				
**Neurological examinations**	110 (65.9)				
Autonomic function testing^b^	36 (21.6)	55 (13.4)	37 (67.3)	15 (27.3)	3 (5.5)
Electromyography	30 (18)	67 (16.3)	56 (83.6)	8 (11.9)	3 (4.5)
Nerve conduction study	39 (23.4)	74 (18.0)	52 (70.3)	10 (13.5)	12 (16.2)
Quantitative sensory testing^c^	30 (18.0)	64 (15.6)	10 (15.6)	49 (76.6)	5 (7.8)
Skin biopsy	51 (30.5)	74 (18.0)	25 (33.8)	45 (60.8)	4 (5.4)
Sudomotor function testing^d^	12 (7.2)	38 (9.2)	13 (34.2)	21 (55.3)	4 (10.5)
Other	24 (14.4)	35 (8.5)	21 (60.0)	14 (40.0)	0
Unspecified*	9 (5.4)				
**Vascular examinations**	55 (32.9)				
Angiography	5 (3.0)	5 (1.2)	4 (80.0)	1 (20.0)	0
Duplex/Doppler ultrasonography	22 (13.2)	34 (8.3)	16 (47.1)	12 (35.3)	6 (17.6)
Echocardiogram	7 (4.2)	7 (1.7)	7 (100)	0	0
Extremity pressure/brachial index	2 (1.2)	3 (0.7)	1 (33.3)	2 (66.7)	0
Thermography	17 (10.2)	51 (12.4)	10 (19.6)	25 (49.0)	16 (31.4)
Other	32 (19.2)	51 (12.4)	34 (66.7)	17 (33.3)	0
Unspecified*	4 (2.4)				
**Imaging studies**	52 (31.1)				
Computed tomography scan	9 (5.4)	9 (2.2)	6 (66.7)	3 (33.3)	0
Electroencephalography	10 (6.0)	10 (2.4)	8 (80.0)	2 (20.0)	0
Fluoroscopy	0	0	0	0	0
Magnetic resonance imaging	24 (14.4)	29 (7.1)	23 (79.3)	6 (20.7)	0
Positron emission tomography scan	1 (0.6)	1 (0.2)	1 (100)	0	0
Ultrasound	6 (3.6)	6 (1.5)	5 (83.3)	1 (16.7)	0
X-ray	23 (13.8)	24 (5.8)	21 (87.5)	3 (12.5)	0
Other	5 (3.0)	5 (1.2)	3 (60.0)	2 (40.0)	0
Unspecified*	2 (1.2)				

Data presented as *n* (%).

*When the laboratory, neurological, vascular or imaging tests were unspecified in the study, the findings were reported to be normal, except for 1 case report reporting abnormal neurological findings.

^a^Immune / autoimmune tests: measuring immunoglobulin (Ig), or antibody, levels in the blood serum.

^b^Autonomic function testing: deep breathing, Valsalva and tilt test with monitoring of heart rate and/or heart rate variability, blood pressure, end tidal CO2, respiratory frequency, and cerebral blood flow velocity.

^c^Quantitative sensory testing: sensory detection and pain thresholds for mechanical and thermal sensations.

^d^Sudomotor function testing: quantitative sudomotor axon reflex rest or electrochemical skin conductance.

#### Treatment approaches

Various combinations of pharmacological and non-pharmacological treatments and related responses were reported across most studies. There were 97 cases of which there was symptom resolution (i.e. complete “relief” or absence of symptoms related to erythromelalgia including pain, redness, edema, and heat, as well as return to normal activities) through procedural interventions with systemic pharmacotherapy (C = 3), procedural interventions (C = 21), psychological approaches (C = 1), and pharmacotherapy (C = 72). Of these resolved cases, only 18 cases had a confirmed genetic mutation (2 *TRPV3* mutations and 16 *SCN9A* mutations). If follow-up was noted, “symptom resolution” was reported 1 week up to 5 years post-treatment. Procedural interventions were reported in 56 studies (Table 4) which primarily included: neural axial blockade (i.e. epidural catheters insertion), infusions, nerve blocks, sympathectomies, and transcutaneous electrical nerve stimulations. Non-pharmacological approaches included avoidance of triggers (e.g. reduce exposure to heat), physiotherapy, psychology, pain rehabilitation programs, and other treatments (Table 4). Pharmacotherapy was reported in 148 studies (Table 4 and Supplementary Table 4). The most common pharmacological classes included adrenergic agonists, antihistamines, beta-blockers, calcium channel blockers, corticosteroids, cyclooxygenase (COX) inhibitors, opioid receptor agonists, sodium channel blocker, and selective serotonin/serotonin-norepinephrine reuptake inhibitors (SSRIs/SNRIs).

**Table 4:** Therapeutic approaches reported in studies.

	Number of studies with reported treatments (S = 167)	Number of cases with reported treatments (C = 411)	Beneficial treatment response (C = 411)			
			No	Some	Resolved	Not reported
**Procedural interventions**	56 (33.5)					
Electric convulsive therapy	1 (0.6)	1 (0.2)	1 (100)	0	0	0
Ganglionectomy	1 (0.6)	1 (0.2)	0	0	1 (100)	0
Infusion^a^	11 (6.6)	11 (2.7)	3 (27.3)	5 (45.5)	2 (18.2)	1 (9.1)
Intrathecal pump^b^	2 (1.2)	5 (1.2)	2 (40.0)	2 (40.0)	1 (20.0)	0
Neural axial blockade^c^	19 (11.4)	21 (5.1)	6 (28.6)	7 (33.3)	6 (28.6)	2 (9.5)
Radio frequency ablation	2 (1.2)	2 (0.5)	0	2 (100)	0	0
Regional block^d^	20 (12)	22 (5.4)	7 (31.8)	10 (45.5)	4 (18.2)	1 (4.5)
Spinal cord stimulator	7 (4.2)	7 (1.7)	0	2 (28.6)	4 (57.1)	1 (14.3)
Stereotactic cryotherapy	1 (0.6)	3 (0.7)	0	0	3 (100)	0
Sympathectomy	11 (6.6)	24 (5.8)	6 (25.0)	14 (58.3)	4 (16.7)	0
Transcranial magnetic stimulation	1 (0.6)	1 (0.2)	0	1 (100)	0	0
Transcutaneous electrical nerve stimulation	5 (3.0)	8 (1.9)	5 (62.5)	3 (37.5)	0	0
**Non-pharmacological approaches**						
Pain rehabilitation programs	2 (1.2)	2 (0.5)	0	0	0	2 (100)
Physical therapy	13 (7.8)	25 (6.1)	3 (12.0)	1 (4.0)	0	21 (84.0)
Psychology	18 (10.8)	30 (7.3)	4 (13.3)	8 (26.7)	1 (3.3)	17 (56.7)
Trigger management	19 (11.4)	19 (4.6)	0	0	0	19 (100)
Other treatment	14 (8.4)	15 (3.6)	4 (26.7)	8 (53.3)	0	3 (20.0)
**Pharmacotherapy**	148 (88.6)					
Angiotensin-converting enzyme inhibitors						
Captopril	–	2 (0.5)	1 (50.0)	1 (50.0)	0	0
Digoxin	–	1 (0.2)	0	1 (100)	0	0
Fosinopril	–	1 (0.2)	0	0	1 (100)	0
Ramipril	–	1 (0.2)	0	1 (100)	0	0
Adrenergic agonists						
Clonidine	–	20 (4.9)	9 (45.0)	5 (25.0)	1 (5.0)	5 (25.0)
Ephedrine	–	3 (0.7)	2 (66.7)	0	1 (33.3)	0
Isoproterenol	–	2 (0.5)	1 (50.0)	1 (50.0)	0	0
Alpha-blockers						
Phenoxybenzamine	–	6 (1.5)	3 (50.0)	2 (33.3)	1 (16.7)	0
Prazosin	–	3 (0.7)	1 (33.3)	2 (66.7)	0	0
AMPA glutamate receptor antagonists						
Perampanel	–	1 (0.2)	0	1 (100)	0	0
Antibiotics						
Amoxicillin	–	1 (0.2)	0	1 (100)	0	0
Cefazolin	–	1 (0.2)	0	1 (100)	0	0
Chlorhexidine	–	2 (0.5)	0	1 (50.0)	0	1 (50.0)
Ciprofloxacin	–	1 (0.2)	0	1 (100)	0	0
Dicloxacillin	–	1 (0.2)	0	1 (100)	0	0
Gentamicin	–	2 (0.5)	0	1 (50.0)	0	1 (50.0)
Hyaluronic acid	–	1 (0.2)	0	1 (100)	0	0
Minocycline	–	1 (0.2)	0	1 (100)	0	0
Mupirocin	–	2 (0.5)	1 (50.0)	0	1 (50.0)	0
Povidone iodine	–	2 (0.5)	0	0	1 (50.0)	1 (50.0)
Ticarcillin	–	1 (0.2)	0	1 (100)	0	0
Unspecified	–	7 (1.7)	3 (42.9)	2 (28.6)	0	2 (28.6)
Antifungals						
Itraconazole	–	2 (0.5)	0	0	1 (50.0)	1 (50.0)
Terbinafine	–	1 (0.2)	0	0	1 (100)	0
Antihistamines						
Cetirizine	–	12 (2.9)	0	3 (25.0)	0	9 (75.0)
Cinnarizine	–	1 (0.2)	0	1 (100)	0	0
Cyproheptadine	–	5 (1.2)	4 (80.0)	1 (20.0)	0	0
Desloratadine	–	4 (1.0)	1 (25.0)	3 (75.0)	0	0
Diphenhydramine	–	3 (0.7)	1 (33.3)	2 (66.7)	0	0
Hydroxyzine	–	4 (1.0)	2 (50.0)	1 (25.0)	0	1 (25.0)
Rupatadine	–	6 (1.5)	1 (16.7)	5 (83.3)	0	0
Unspecified	–	15 (3.6)	11 (73.3)	4 (26.7)	0	0
Beta-blockers						
Unspecified	–	11 (2.7)	9 (81.8)	1 (9.1)	0	1 (9.1)
Atenolol	–	3 (0.7)	1 (33.3)	1 (33.3)	0	1 (33.3)
Labetalol	–	3 (0.7)	0	1 (33.3)	1 (33.3)	1 (33.3)
Metoprolol	–	1 (0.2)	0	0	1 (100)	0
Nebivolol	–	1 (0.2)	0	0	1 (100)	0
Propranolol	–	13 (3.2)	8 (61.5)	3 (23.1)	1 (7.7)	1 (7.7)
B-tubulin polymerization inhibitors						
Colchicine	–	4 (1.0)	3 (75.0)	1 (25.0)	0	0
Calcium channel blockers						
Amlodipine	–	3 (0.7)	1 (33.3)	2 (66.7)	0	0
Gabapentin	–	86 (20.9)	28 (32.6)	29 (33.7)	5 (5.8)	24 (27.9)
Nifedipine	–	11 (2.7)	8 (72.7)	1 (9.1)	0	2 (18.2)
Nimodipine	–	1 (0.2)	0	1 (100)	0	0
Pregabalin	–	29 (7.1)	12 (41.4)	7 (24.1)	1 (3.4)	9 (31.0)
Cholinergic antagonists						
Procyclin	–	1 (0.2)	0	1 (100)	0	0
Corticosteroids						
Dexamethasone	–	1 (0.2)	0	0	1 (100)	0
Hydrocortisone	–	1 (0.2)	0	0	1 (100)	0
Methylprednisolone	–	9 (2.2)	4 (44.4)	3 (33.3)	2 (22.2)	0
Prednisolone	–	6 (1.5)	1 (16.7)	2 (33.3)	2 (33.3)	1 (16.7)
Prednisone	–	15 (3.6)	1 (6.7)	5 (33.3)	4 (26.7)	5 (33.3)
Unspecified	–	40 (9.7)	25 (62.5)	13 (32.5)	0	2 (5)
COX inhibitors						
Acetaminophen	–	31 (7.5)	14 (45.2)	5 (16.1)	0	12 (38.7)
Aspirin / Acetylsalicylic acid	–	127 (30.9)	84 (66.1)	19 (15.0)	8 (6.3)	16 (12.6)
Celecoxib	–	2 (0.5)	0	2 (100)	0	0
Diclofenac	–	3 (0.7)	2 (66.7)	0	1 (33.3)	0
Indomethacin	–	8 (1.9)	5 (62.5)	2 (25.0)	1 (12.5)	0
Ketorolac	–	4 (1.0)	3 (75.0)	1 (25.0)	0	0
Naproxen	–	10 (2.4)	7 (70)	1 (10)	0	2 (20)
Piroxicam	–	1 (0.2)	0	0	1 (100)	0
Prostaglandin	–	2 (0.5)	0	1 (50.0)	1 (50.0)	0
Sulindac	–	1 (0.2)	0	0	1 (100)	0
Unspecified NSAIDs	–	39 (9.5)	25 (64.1)	4 (10.3)	0	10 (25.6)
Dopamine and/or serotonin agonists						
Risperidone	–	6 (1.5)	0	0	1 (16.7)	5 (83.3)
Chlorpromazine	–	4 (1.0)	1 (25.0)	3 (75.0)	0	0
Cyclobenzaprine	–	1 (0.2)	0	1 (100)	0	0
Haloperidol	–	2 (0.5)	1 (50.0)	0	1 (50.0)	0
Ketanserin	–	1 (0.2)	0	1 (100)	0	0
Methylsergide	–	5 (1.2)	3 (60)	0	2 (40)	0
Pizotifen	–	2 (0.5)	1 (50.0)	0	1 (50.0)	0
EGFR blockers						
Erlotinib	–	3 (0.7)	0	0	3 (100)	0
Emollients						
Glycyrrhizin	–	2 (0.5)	1 (50.0)	0	1 (50.0)	0
GABA-A receptor agonists						
Brotizolam	–	2 (0.5)	0	2 (100)	0	0
Chloral hydrate	–	2 (0.5)	0	1 (50.0)	0	1 (50.0)
Clobazam	–	1 (0.2)	0	0	1 (100)	0
Clonazepam	–	12 (2.9)	6 (50.0)	0	2 (16.7)	4 (33.3)
Diazepam	–	7 (1.7)	4 (57.1)	1 (14.3)	0	2 (28.6)
Lorazepam	–	3 (0.7)	0	1 (33.3)	0	2 (66.7)
Midazolam	–	2 (0.5)	1 (50.0)	1 (50.0)	0	0
Unspecified sedatives	–	7 (1.7)	4 (57.1)	1 (14.3)	0	2 (28.6)
Epidermal growth factors	–	2 (0.5)	0	1 (50.0)	1 (50.0)	0
Immunoglobulins	–	8 (1.9)	2 (25.0)	5 (62.5)	1 (12.5)	0
Immunosuppressants						
Cyclosporine	–	2 (0.5)	1 (50.0)	0	1 (50.0)	0
Hydroxychloroquine	–	1 (0.2)	0	0	1 (100)	0
Tacrolimus	–	1 (0.2)	0	1 (100)	0	0
Medicinal plants	–	2 (0.5)	0	0	2 (100)	0
Melatonin	–	1 (0.2)	0	0	1 (100)	0
Nitric oxides						
Nitroglycerin	–	4 (1.0)	3 (75.0)	1 (25.0)	0	0
NMDA antagonists						
Ketamine	–	11 (2.7)	3 (27.3)	4 (36.4)	0	4 (36.4)
Opioid receptor agonists						
Buprenorphine	–	1 (0.2)	0	0	1 (100)	0
Codeine	–	7 (1.7)	3 (42.9)	1 (14.3)	0	3 (42.9)
Fentanyl	–	4 (1.0)	1 (25.0)	1 (25.0)	0	2 (50.0)
Hydromorphone	–	4 (1.0)	1 (25.0)	1 (25.0)	0	2 (50.0)
Methadone	–	7 (1.7)	3 (42.9)	1 (14.3)	0	3 (42.9)
Morphine	–	19 (4.6)	9 (47.4)	6 (31.6)	0	4 (21.1)
Oxycodone	–	7 (1.7)	4 (57.1)	1 (14.3)	0	2 (28.6)
Tramadol	–	16 (3.9)	9 (56.3)	2 (12.5)	0	5 (31.3)
Unspecified opiates	–	28 (6.8)	10 (35.7)	10 (35.7)	0	8 (28.6)
Platelet activation inhibitors						
Nafazatrom	–	1 (0.2)	0	1 (100)	0	0
Nitroprusside	–	18 (4.4)	8 (44.4)	1 (5.6)	8 (44.4)	1 (5.6)
Sex hormone agonists						
Noretistherone	–	1 (0.2)	0	1 (100)	0	0
Sodium channel blockers						
Bupivacaine	–	1 (0.2)	0	0	1 (100)	0
Carbamazepine	–	52 (12.7)	12 (23.1)	16 (30.8)	3 (5.8)	21 (40.4)
Lacosamide	–	1 (0.2)	0	1 (100)	0	0
Lidocaine patch	–	27 (6.6)	4 (14.8)	9 (33.3)	2 (7.4)	12 (44.4)
Lidocaine	–	33 (8.0)	9 (27.3)	14 (42.4)	3 (9.1)	7 (21.2)
Mexiletine hydrochloride	–	64 (15.6)	8 (12.5)	17 (26.6)	20 (31.3)	19 (29.7)
Oxcarbazepine	–	11 (2.7)	3 (27.3)	3 (27.3)	0	5 (45.5)
Pramocaine	–	1 (0.2)	0	0	1 (100)	0
Investigative sodium channel blocker	–	7 (1.7)	0	7 (100)	0	0
SSRIs/SNRIs						
Amitriptyline	–	39 (9.5)	21 (53.8)	12 (30.8)	1 (2.6)	5 (12.8)
Clomipramine	–	1 (0.2)	0	1 (100)	0	0
Duloxetine	–	8 (1.9)	4 (50.0)	3 (37.5)	1 (12.5)	0
Escitalopram	–	3 (0.7)	0	1 (33.3)	0	2 (66.7)
Fluvoxamine	–	1 (0.2)	0	1 (100)	0	0
Imipramine	–	1 (0.2)	0	1 (100)	0	0
Nortriptyline	–	4 (1.0)	3 (75.0)	1 (25.0)	0	0
Paroxetine	–	3 (0.7)	0	1 (33.3)	1 (33.3)	1 (33.3)
Sertraline	–	6 (1.5)	0	3 (50.0)	0	3 (50.0)
Venlafaxine	–	10 (2.4)	4 (40.0)	5 (50.0)	0	1 (10.0)
Unspecified	–	22 (5.4)	9 (40.9)	5 (22.7)	0	8 (36.4)
Supplements						
Iron	–	1 (0.2)	0	1 (100)	0	0
Magnesium	–	7 (1.7)	2 (28.6)	3 (42.9)	2 (28.6)	0
Vitamin B complex	–	1 (0.2)	0	0	1 (100)	0
TNF-alpha inhibitors						
Adalimumab	–	1 (0.2)	0	0	1 (100)	0
Topical compounds	–	14 (3.4)	3 (21.4)	4 (28.6)	2 (14.3)	5 (35.7)
TRPV1 agonists						
Capsaicin	–	8 (1.9)	4 (50.0)	2 (25.0)	2 (25.0)	0
Wound dressings	–	2 (0.5)	0	0	2 (100)	0
Unspecified analgesics	–	25 (6.1)	7 (28.0)	1 (4.0)	0	17 (68.0)
Unspecified epileptics	–	10 (2.4)	5 (50.0)	1 (10.0)	0	4 (40.0)
Unspecified vasodilators	–	18 (4.4)	7 (38.9)	2 (11.1)	0	9 (50.0)

Data presented as *n* (%).

“Some” beneficial treatment response included minor to major improvement in patient symptoms, while “resolved” cases represented complete relief.

*AMPA* α-amino-3-hydroxy-5-methyl-4-isoxazolepropionic acid, *COX* cyclooxygenase, *EGFR* epidermal growth factor receptor, *GABA* gamma-aminobutyric acid, *NMDA* N-methyl-D-aspartate, *NSAID* non-steroidal anti-inflammatory drug, *SSRI* Selective serotonin reuptake inhibitor, *SNRI* Serotonin and norepinephrine reuptake inhibitor, *TNF* Tumor necrosis factor, *TRPV* Transient receptor potential channels of the vanilloid subtype.

^a^Infusions primarily included a sodium channel blocker (e.g. bupivacaine, lidocaine), an NMDA antagonist (ketamine), and/or a platelet activation inhibitor (nitroprusside).

^b^Only one case included details from their intrathecal pump which included an opioid receptor agonist (morphine), a calcium channel antagonist (Ziconotide), and an unspecified anesthetic.

^c^Epidural cathethers primarily included a sodium channel blocker (e.g. bupivacaine, lidocaine, lignocaine) with or without an opioid receptor agonist (e.g. morphine, fentanyl).

^d^Nerve blocks included brachial plexus, lower extremity peripheral, lumbar caudal, lumbar epidural, lumbar sympathetic, sciatic, and tibiofibular nerve blocks.

## Discussion

To our knowledge, this is the first scoping review to map the existing literature in pediatric erythromelalgia. Our goal was to identify gaps in knowledge to inform future research. A total of 167 studies totalling 411 individuals with childhood-onset erythromelalgia were identified. Findings suggest contrasting clinical presentations, assessment, and treatment of pediatric erythromelalgia.

**Fig. 1:**
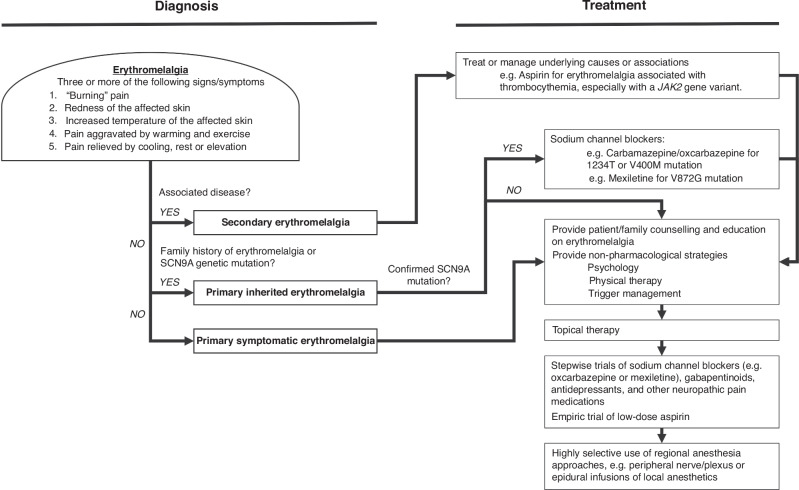
Proposal for diagnostic workup and classification for pediatric erythromelalgia and treatment approach. Burning pain, redness, and increased temperature of the affected skin can be either continuous or episodic.

Only 53 studies reported on all five of Brown’s 1932 diagnostic criteria in their cases, representing 40% of the cases.^2^ This illustrates the significant variability in clinical presentation of pediatric erythromelalgia cases, highlighting its diagnostic challenge. Case-control studies and clinical trials identified were not usually specific in their inclusion criteria^17–19^ such as “documented diagnosis of erythromelalgia, as characterized by redness, warmth, and burning pain of the extremities (most commonly feet), typically precipitated by heat or exercise and relieved by cooling.”^20^ Moreover, cases have reported their main affected areas as the face, ears or groin, which are not usually included in the definition of extremities, or have reported their symptoms as episodic or continuous. Future directions should test the sensitivity and specificity of diagnostic criteria of EM, and whether there are differences for those who present symptoms during the pediatric lifespan. We propose at least three of five criteria based on Brown’s diagnostic criteria must be met for a presumptive diagnosis of erythromelalgia (episodic or continuous) (Fig. 1). Diagnosing erythromelalgia in youth is a critical step towards its recognition and validation, and may lead to its treatment and the opportunity for the patient to connect with support groups (e.g. The Erythromelagia Association, The Erythromelalgia Warriors, Ben’s Friends - Living with Erythromelalgia online community, etc.) and identify self-management strategies.

Contrasting hypotheses on the pathophysiology of pediatric erythromelalgia have led to widely varying treatment approaches. Tham et al. conducted a critical review of current pain management approaches for patients with erythromelalgia and concluded there were no established best practices or clinical guidelines for pain management in this disorder.^7^ Similar to approaches identified in our scoping review, management included avoiding situations that may precipitate pain, as well as incorporating pharmacotherapy, procedural interventions, and non-pharmacological interventions.^7,12,13^ Qualitatively, findings from our scoping review highlight that the current treatment of pediatric erythromelalgia is a stepwise trial and error approach. Ma et al. published a proposed approach to management including non-pharmacological and pharmacological (topical and systemic) strategies.^21^ They highlight that labelling erythromelalgia as secondary or primary would not affect the treatment approach significantly. However, several specific subtypes have been identified for which making a mechanistic diagnosis can lead to more targeted treatment.^18–20,22–25^ Based on these reports, we propose a modified approach for youth with erythromelalgia (Fig. 1).

Upon evaluation, it is important to treat or manage any underlying causes or associations (e.g. myeloproliferative diseases). Laboratory tests and imaging studies for most cases reported in this scoping review were primarily used to exclude secondary diseases associated with erythromelalgia. The scoping review highlights the preponderance of negative findings from laboratory tests and imaging studies in youth with erythromelalgia. As an example, testing for Fabry disease, where supported by features of the history and exam, has therapeutic importance because of the availability of a specific enzyme replacement therapy.^26,27^ However, among the cases and unpublished experience of the co-authors, Fabry disease is likely to account for only a very small fraction of patients presenting with erythromelalgia. Neurological and vascular examination findings were consistent with literature in adult populations. There was a small proportion with abnormal findings of increased blood flow and temperature, or large and/or small fiber neuropathy.^28,29^ The diverse and limited positive findings highlight the overall heterogeneity in clinical presentation of erythromelalgia which has led to contrasting views of its underlying pathophysiology (neurologic, inflammatory, autoimmune, vascular, etc.). Erythromelalgia associated with thrombocythemia has been linked to gene variants producing a constitutively activated form of Janus Kinase 2 (JAK2).^22^ Essential thrombocythemia is one of many myeloproliferative disorders whose symptoms may include red, warm, burning, or tingling hands or feet. Patients with erythromelalgia with associated thrombocythemia have displayed relief by aspirin due to its irreversible inhibition of platelet cyclooxygenase activity.^22^ However, the lack of longitudinal trajectories of youth with erythromelalgia and low prevalence of high platelet counts from available patient samples argues against the hypothesis that thrombocythemia is a frequent cause of erythromelalgia.^30^

Oaklander has postulated that erythromelalgia may be driven primarily through small-fiber neuropathy associated with systemic autoimmune/autoinflammatory disorders.^31^ Conventional electrodiagnostic tests such as electromyography or nerve conduction studies are insensitive to small-fiber neuropathy. Skin biopsies are safe and minimally invasive, and have been recommended by Oaklander and others as useful in diagnosis of small-fiber neuropathy. Case reports of erythromelalgia with small-fiber neuropathy have reported improvements after intravenous immunoglobulin therapy or corticosteroids.^32,33^ However, there are limited reference values for skin neurite densities in healthy children, and there are uncertainties about how to interpret positive and negative predictive values.^34–36^ A randomized controlled trial in 30 adults with painful idiopathic small fiber neuropathy found that intravenous immunoglobulin treatment had no significant effect on pain compared to placebo control.^37^ Our scoping review highlights that clinicians have limited information to guide them on how best to use clinical variables to prioritize laboratory testing for other diseases that could present as erythromelalgia. Future consensus is warranted to determine the diagnostic procedure for youth displaying the signs and symptoms suggestive of erythromelalgia.

In 2004, several cases of Mendelian inherited erythromelalgia were linked to autosomal dominant gain-of-function mutations of the *SCN9A* gene. *SCN9A* encodes for the voltage-gated sodium channel Na_v_1.7, which is found primarily in small peripheral sensory and sympathetic neurons.^16^ Gain-of-function mutations in *SCN9A* lead to an increase in cellular sodium influx resulting in increased signaling in nociceptive neurons. In a recent systematic review, Arthur et al. identified 16 different substitutions of Na_v_1.7 channels,^5^ while up to 32 *SCN9A* gene variants have now been identified in 146 cases in our scoping review. The increase in novel *SCN9A* gene variants can be attributed to an increase in recognition/publication of erythromelalgia, advancement in genetic analyses, and growth of genetic databases. For patients with *SCN9A* variants, studies suggest that the electrophysiological properties of the specific variant channel may predict responsiveness to sodium channel blockers such as carbamazepine, oxcarbazepine, and mexiletine.^23–25^ However, in our scoping review, mutations of the *SCN9A* gene were identified as the cause of only 35% of reported pediatric cases of erythromelalgia. Therefore, the majority of cases may have non-genetic etiologies or a yet unidentified mutation in one or more genes. Plausible candidates could be genes involved upstream or downstream of Na_v_1.7 activity or previously implicated in neurological, inflammatory, vascular, or pain disorders,^38–40^ such as Familial Episodic Pain Syndrome (*TRPA1*).^41^ Adult erythromelalgia case reports have identified genetic variants that alter the function of other voltage-gated sodium channels (*SCN10A* and *SCN11A*),^42^ or platelet-endothelial interactions (e.g. *JAK2*),^22^ as noted above. The identification of novel variants related to erythromelalgia will have broader implications in understanding pain mechanisms in general and may hopefully lead to novel approaches to pain treatment, as previously done with Na_v_1.7.^43^

Based on the findings of this scoping review, the levels of evidence for treatment for youth with erythromelalgia are considered low. Topical treatments are usually considered as a first-line pharmacological treatment, since they cause fewer adverse effects compared to systemic medications and interventional procedures.^21^ In our scoping review, medications used for neuropathic pain, including antidepressants, anticonvulsants, and sodium channel blockers were commonly prescribed for youth with erythromelalgia. Evidence for these drug classes for neuropathic pain in children is sparse,^44^ and prescribing is largely based on extrapolation from adult studies of other forms of neuropathic pain. While gabapentin is often the first medication prescribed for neuropathic pain in other settings, it is the practice of several of the authors of this review to select a sodium channel blocker, e.g. oxcarbazepine or mexiletine, as the first neuropathic medication for a trial in children with erythromelalgia.

When underlying causes or associations cannot be determined, it is important to provide counselling and non-pharmacological strategies addressing psychosocial factors involved with erythromelalgia. Psychosocial factors and quality of life were reported in only 65 and 211 cases, respectively. Recognizing that pain is a complex multidimensional experience that is the result of interactions between biological, psychological and social factors,^45^ highlights the need to study the perspective and experiences of youth with erythromelalgia. Our scoping review highlights significant co-morbidities in youth with erythromelalgia which include anxiety, depression, suicidal ideation, and sleep impairment, alongside physical and social limitations. Despite the small proportion of cases reporting mental health comorbidities, studies focusing on its co-occurrence with pediatric chronic pain have shown higher mental health issues in youth with chronic pain compared to their pain-free peers.^46^ Recognizing the prevalence of these factors and addressing them with a multidisciplinary team that includes mental health providers is essential for these cases. Therefore, future research should incorporate core domains and outcome measures^47^ for pediatric chronic pain trials and registries that encompass measures of pain severity, pain-related interference, overall well-being, emotional and physical functioning, and sleep.^48,49^

Referral to a comprehensive multidisciplinary pain rehabilitation center could be considered for youth with severe, refractory, or disabling EM. However, whether exercise exacerbates the symptoms of erythromelalgia is important to consider for rehabilitation, especially as this was reported for nearly 60% of the cases in this scoping review. Nevertheless, understanding the pathophysiology of pediatric erythromelalgia and identifying clinical subgroups within a large sample of pediatric erythromelalgia patients may offer an initial step to determining individualized therapeutic approaches.

Most of the reports in this scoping review were retrospective in nature. This has inherent limitations, as the data are primarily drawn from text fields with descriptive or narrative responses, and there is missing data. Structured and/or standardized inputs for diagnostic criteria will be beneficial for future research purposes. As pediatric erythromelalgia is a rare condition, the development of an international registry would immensely benefit multidisciplinary experts involved in the care of pediatric erythromelalgia and those with lived experience. In this regard, our team is developing a multicenter PEDiatric ErythoMElalgia Registry Gathering multidisciplinary Experts (PED-EMERGE) to investigate our hypothesis that erythromelalgia is a clinical syndrome that includes multiple mechanisms in distinct patient subgroups. Moreover, our team recruits pediatric erythromelalgia patients to undergo genetic screening which may lead to the discovery of new Mendelian causes of erythromelalgia or predisposing genes that are conserved across pediatric cases. Our team includes patient partners involved in ensuring research projects and the registry are patient-centered. The objective of this consortium is to create collaborations between diverse experts and generate patient-centered clinical effectiveness research projects.

This scoping review revealed variability in the clinical presentation of pediatric erythromelalgia regarding diagnostic criteria, clinical examination findings and treatments offered. Ongoing efforts focus on developing a multicenter registry to standardize data collection and reporting with the goal of establishing consensus recommendations for the diagnosis and management of pediatric erythromelalgia.

## Supplementary information

Supplementary Material

## Data Availability

All articles included the data extraction are included in the Supplementary Material.
