# Supplementary material for: Pediatric erythromelalgia from multidisciplinary perspectives: a scoping review
Source: Pediatr Res. 2025 Jan 16;98(3):786–99. doi: 10.1038/s41390-025-03817-4 (PMC12507648; doi:10.1038/s41390-025-03817-4)
Supplement: Supplementary file 1 — Supplementary Material [file 41390_2025_3817_MOESM1_ESM.pdf]

Supplementary Material

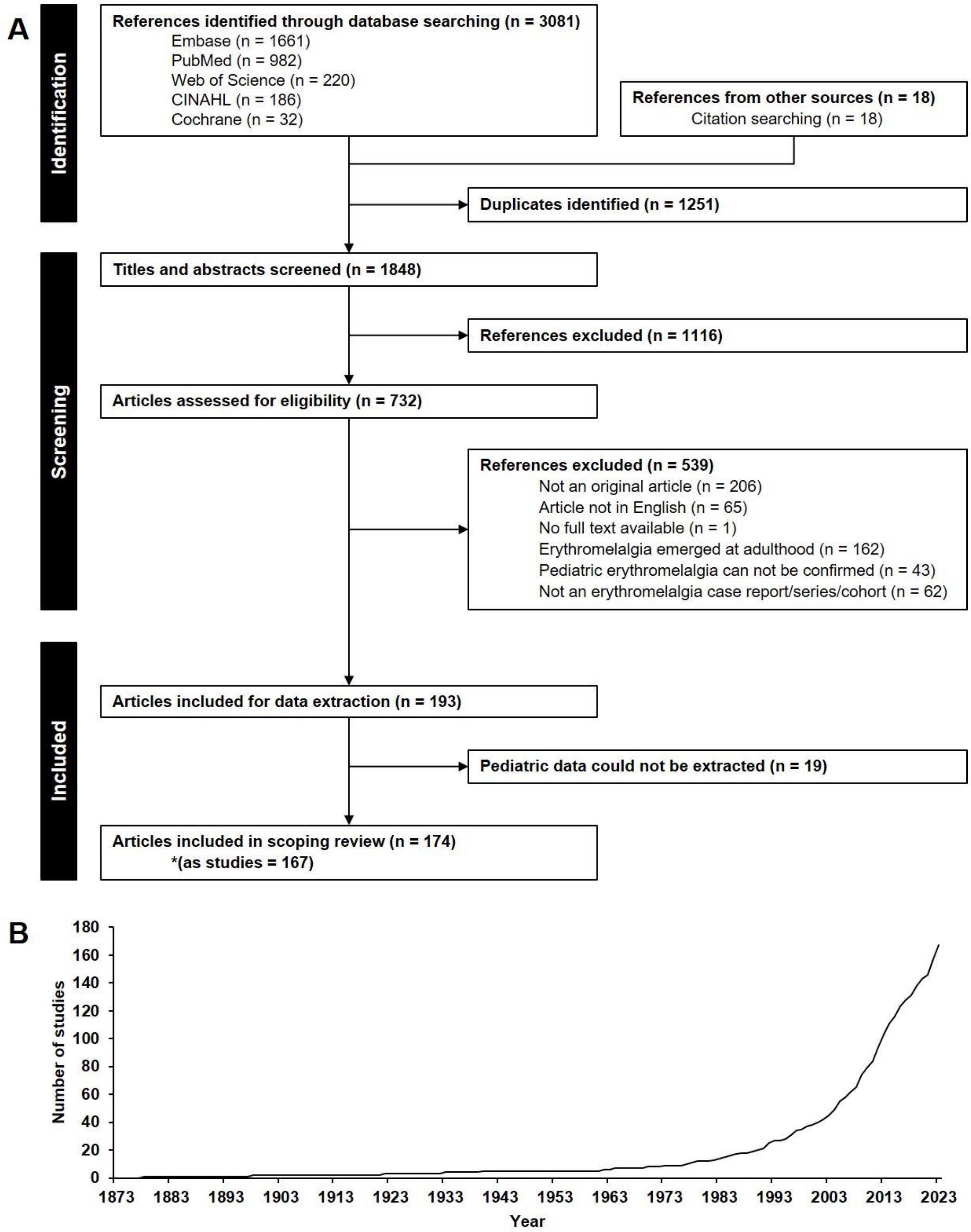

Supplementary Figure 1. A) Preferred Reporting Items for Systematic Reviews and Meta-Analyses extension for Scoping Reviews flow diagram. *Overlap in case reports in the 174 articles led to 167 studies overall. B) Cumulative number of studies including pediatric erythromelalgia cases over time

Supplementary Table 1. Summary of studies with only aggregate data including pediatric patients

Authors	Year	PMID	Main findings
Babb et al.	1964	14105024	While pediatric patients were not reported separately from adult patients, information about the distribution of primary versus secondary erythermalgia can be valuable in establishing treatment. Of 51 patients in this study, 30 were identified as having primary erythermalgia because of the absence of associated conditions, while 21 were identified as having secondary erythermalgia because the condition was associated with various other diseases. Of note, the associated disease in secondary erythromelalgia was a type of myeloproliferative disorder in 10 of the 21, although erythermalgia preceded other symptoms of the myeloproliferative disorder by as long as 12 years in some cases. Primary erythromelalgia was found more often in younger patients and seemed to present more severely, producing greater pain that was more often bilateral and involved larger areas of the affected extremities.
Zheng et al.	1991	1785945	Erythromelalgia outbreaks as an epidemic disease have generally occurred during the winter or spring every 3-5 years in Southern China since 1954. Patients with the disease have shown a poxvirus, named erythromelalgia related poxvirus, which seems to have an etiological role in epidemic erythromelalgia.
Kalgaard et al.	1997	9350163	Etiology, severity, and prognosis of erythromelalgia seem to be heterogeneous amongst patients, and lower severity cases may be more common that previously thought. This clinical study of 87 cases argued that erythromelalgia should not be considered as a separate disease entity, but should rather be considered to be caused by microvascular arterio-venous shunting. Primary erythromelalgia seems to be the most common subgroup of this heterogeneous condition, and an autosomal dominant mode of heredity is suspected. Primary erythromelalgia patients also seem to be younger than secondary erythromelalgia patients, which is relevant for investigating pediatric cases in particular.
Kvernebo	1998	000077105700001*	The aim of this report is to present a hypothesis of the pathogenic mechanisms of erythromelalgia, based on a review of the literature, and the results of clinical, epidemiological, histopathological, and pathophysiological studies of 40 patients with erythromelalgia. A new clinical system for grouping patients is proposed.
Sandroni et al.;<br>Davis et al.	1999;<br>2000	10724194;<br>19078553	The goal of this study, which included 168 patients, was to describe demographics, presentation, and outcome in patients with erythromelalgia. The sample was entirely white and 72% female, with 7 participants who had symptoms since childhood. Symptoms were predominantly in feet (148/168) and hands (43/168) and predominantly intermittent, although they were constant in 5 participants. After a mean follow-up of 8.7 years, only 29 participants reported improvement and only 10 complete resolution of symptoms, while the rest reported either worsening or no change in symptoms. Kaplan-

			Meier survival curves showed a significant decrease in survival in erythromelalgia patients compared with the expectation for persons of similar age and same sex. All of this indicated that erythromelalgia has a significantly increased mortality and morbidity compared with the general US population and improved treatment is necessary.
Mork et al.	2000	10733667	By examining patients and controls before and after central body heating with laser Doppler perfusion imaging, this study showed support for their hypothesis that erythromelalgia symptoms develop as a result of arteriovenous blood shunting in the skin, which leads to hypoxia. The researchers believe that tissue oxygen tension varies considerably in patients with symptomatic primary erythromelalgia, with high values near perfused vessels and low values in other areas.
Kalgaard et al.	2003	14690340	Iloprost seemed to have a treatment effect on erythromelalgia, reducing both the sympathetic dysfunction and the need for cooling of the affected skin. Reductions in flux after Valsalva's maneuver and contralateral cooling were significantly higher one month after treatment with iloprost compared to baseline. These data indicated that prostacyclin analogues like iloprost could be viable treatments for erythromelalgia and that a larger placebo-controlled study of these analogues would be beneficial to the field.
Kazemi et al.	2003	12712804	This study's findings confirmed previous work about skin biopsy of these patients, and it revealed sympathetic neuropathy or small C fiber axon involvement in erythromelalgia. 72.7% of participants showed abnormal sympathetic skin response (SSR) in lower limbs, and there was a significant difference between primary erythromelalgia patients and the control group for SSR in lower limbs. Sympathetic neuropathy in erythromelalgia should be further studied.
Ørstavik et al.	2003	12566278	This study was the first to report systematic recordings from pathological C-nociceptive fibres in patients with a chronic pain condition. The data suggests that impulse propagation is altered in afferent, but not efferent, C-fibers, with lower conduction velocity and a more pronounced activity dependent slowing, which suggests incipient afferent small-fiber C-fibers in chronic pain neuropathy. Mechanical sensitization and spontaneous activity in originally mechano-insensitive C-fibers correlates with the symptoms in the patients and suggests an active role of these 'sleeping nociceptors' in erythromelalgia.
Davis et al.	2006	17178984	Thermoregulatory sweat testing (TST) is one method of testing small-nerve fiber function which often correlates with abnormal findings in other tests of autonomic or small-fiber functions, and it may aid in the diagnosis of underlying small-fiber neuropathy in patients with erythromelalgia. As this study showed abnormal TST results in 28/32 (88%) patients with erythromelalgia, the authors believe it to be likely that small-fiber neuropathy is one possible cause of erythromelalgia.

Davis et al.	2006	16924077	Although Raynaud phenomenon and erythromelalgia seem to be completely opposite conditions, it is possible that they may have similar causes and underlying pathogenic mechanisms. Two thirds of the patients in this study showed acrocyanosis, Raynaud phenomenon, and/or cool blue extremities between episodes of erythromelalgia, which suggests that the changes to each extreme occur along a continuum of vascular instability. These authors proposed that observed small-fiber neuropathy plays a significant role in this vascular instability, contributing both to erythromelalgia episodes and to symptoms more reminiscent of Raynaud phenomenon.
Reed & Davis	2009	18713229	The data from this study showed that the incidence of erythromelalgia seems to be increasing with both time and age, which is consistent with a previous study. The authors did not believe that this increase is due to a recent recognition of erythromelalgia, as physicians in Olmsted County, Minnesota, have been observing the disease for several decades.
Genebriera et al.	2012	22369570	Multiple sensory modalities were found to be abnormal in patients with erythromelalgia, with the most common clinical abnormality being isolated heat and pain abnormality. These findings supported the notion that neuropathy underlies the clinical diagnosis of erythromelalgia. Future studies should explore the nature of the relationship between these sensory abnormalities and the clinical features of erythromelalgia.
Poterucha et al.	2013	23545913	This study evaluated patients with erythromelalgia who were prescribed compounded topical amitriptyline-ketamine from 2004-2011. The majority (75%) of patients with erythromelalgia reported reduced pain after the use of a topical treatment of amitryptyline and ketamine. This topical treatment seems to be a feasible method to treat pain in erythromelalgia, but it is unclear if it is equally effective in adult and pediatric patients.
Davis	2013	23768296	For patients with recalcitrant erythromelalgia, it was important to consider whether the condition is complicated by repeated exposure to cold and/or water, because prevention of the inciting behaviors can resolve the clinical manifestation. Addressing these behaviors could help manage and prevent worsening of erythromelalgia.
Zhang et al.	2014	29911575	Functionally characterized mutations of NaV1.7 (I848T) are present only in a minority of patients with erythromelalgia. Although more than half of the patients in this cohort (27/48) carried rare protein-modifying mutations, the majority of those will probably not be causally linked to erythromelalgia. Future research should investigate the possible role of rare variants of NaV1.8, NaV1.9, or beta-subunits in provoking chronic pain conditions or erythromelalgia.

Mantyh et al.,	2017	27926760	While only 5 of 52 patients in this cohort had epidermal nerve fiber density (ENFD) at or below the fifth percentile of healthy control individuals, most patients had functional abnormalities of these small fibers, 29 patients had abnormal sweat test results, 21 had abnormal pain thresholds, and 20 had abnormal blood pressure or heart rate control. Unlike other diseases of the small nerve fibers that cause acral pain syndromes, erythromelalgia is not characterized by loss of ENFD, although most patients do have impaired function of these small fibers. For this reason, physicians should perform functional rather than structural small fiber studies when evaluating erythromelalgia.
Pagani-Estevez et al.	2017	28413058	This study aimed to identify cases of erythromelalgia that were responsive to treatment with corticosteroids. Of the 31-patient cohort, 14 did not respond to corticosteroids, 8 responded partially, and 9 responded completely. 6 of the participants who experienced complete corticosteroid response reported a disease precipitant of some kind, such as surgery, trauma, or infection, and steroid responders also received the steroid earlier in disease progression than steroid non-responders (3 vs 24 months). This data suggested that an infectious, traumatic, or surgical precipitant and subacute presentation may portend corticosteroid respondent epidermal nerve fiber density, and there may be a transient window of time where corticosteroid is useful before irreversible nociceptive remodeling and central sensitzation occurs.
Parker et al.	2017	27494156	This study suggested that erythromelalgia is more common in women, with a large age spread and wide variation of symptoms, although sensations of burning and heat were found in most patients. Consistent with other studies, patients found that their symptoms were worse in hot environments and relieved with cooling. The findings of this study confirm the refractory nature of erythromelalgia and its resistance to many treatment options. The best response seemed to be with intravenous prostacyclin therapy, which was described as somewhat or very helpful by 63% of patients. This approach should be considered in severe or refractory cases.

*Web of Science ID

Supplementary Table 2. References included in this scoping review		
**Authors**	**Year**	**PMID**
Mitchell	1878	NLM: 101660182
Collier	1898	DOI: 10.1016/S0140-6736(01)99580-7
Stott & Dore	1922	19982254
Lewis	1933	N/A
Telford & Simmons	1940	20783429
Cross	1962	13882378
Catchpole	1964	14124085
Burbank et al.;<br>Finley et al.	1966;<br>1992	WOS:A19668468900033;<br>1536168
Jelinek	1970	5427446
Alarcon-Segovia & Diaz-Jouanen	1973	4742370
Mandell et al.	1977	840540
Jorgensen & Sondergaard	1978	619769
Thomson et al.;<br>Samuels et al.	1978;<br>2008	535232;<br>18518989
Ozsoylu et al.	1979	430305
Cohen & Samorodin	1982	7138057
Uno & Parker	1983	6336930
Ozsoylu & Coskun	1984	6698066
Thomas	1985	4078894
Kinsella & Irwin	1986	3804826
Kvernebo & Seem	1987	3559792
Michiels et al.	1989	2808845
Kandel	1990	2080386
Calderone & Finzi	1991	1999514
Chakravarty et al.	1992	1561190
D'Angelo et al.	1992	1734777
Strozik et al.	1992	1628475
Herskovitz et al.	1993	8451013
Wagner et al.	1993	8442534
Drenth et al.	1995	8582398
Drenth et al.	1996	8721588
Krishnan et al.	1996	20948053
Rauck et al.	1996	8610879
Confino et al.	1997	9093785
McGraw & Kosek	1997	9105243
Stone et al.	1997	9161655
Kasapcopur et al.	1998	9536398
Kuhnert et al.	1999	10606048
Rabaud et al.	1999	9918272
Brar et al.	2000	DOI: 10.1163/156856900750228132
Cimaz et al.	2001	11476842
Saviuc et al.	2001	11527236
Chan et al.	2002	12193436
Davis & Sandroni	2002	11790162
Davis et al.	2003	14568838
Harrison et al.	2003	12495966
Sano et al.	2003	12930941
Dell'Era et al.	2004	N/A
Jang et al.	2004	15377366
Ørstavik et al.	2004	15288393
Yang et al.	2004	14985375

Davis & Sandroni	2005	16230578
Delye et al.	2005	16234658
Dib-Hajj et al.	2005	15958509
Michiels et al.	2005	16216943
Nathan et al.	2005	15741349
Paira et al.	2005	16357758
Han et al.	2006	16392115
Harty et al.	2006	17135418
Sandroni & Davis	2006	16549702
Misery et al.	2007	17958876
Novella et al.	2007	17410110
Takahashi et al.	2007	17985268
Zhang et al.	2007	17263810
Lee et al.;<br>Cheng et al.	2007;<br>2008	17294067;<br>18171466
Drenth et al.	2008	18347287
Choi et al.	2009	19162012
Fischer et al.	2009	19557861
Galimberti et al.	2009	19368608
Han et al.	2009	19369487
Iqbal et al.	2009	19584578
Li et al.	2009	19456783
Natkunarajah et al.	2009	19549232
Pfund et al.	2009	19056270
Seneschal et al.	2009	19533204
Ahn et al.	2010	20385509
Bang et al.	2010	20552075
Firinci et al.	2010	21053826
Lipsker	2010	20925548
Zhang et al.	2010	20959280
Cheng et al.	2011	21705421
Faddoul	2011	21417202
Johnson et al.	2011	21541228
McElhiney	2011	N/A
Yuan & He	2011	21421200
Cook-Norris et al.	2012	21798623
Goldberg et al.	2012	22035805
Grandy et al.	2012	23008794
Jakob et al.	2012	22170168
Moody et al.	2012	22156790
Morales et al.	2012	22718865
Prabhu et al.	2012	23211921
Segerdahl et al.	2012	22365309
Skeik et al.	2012	22033523
Yi et al.	2012	22134130
Cregg et al.	2013	23292638
Elgueta et al.	2013	23368881
Estacion et al.	2013	23376079
Kalava et al.	2013	23872544
Kim et al.	2013	23152140
Klein et al.	2013	23129781
Messeguer et al.	2013	23473286
Wu et al.	2013	22612394
Wu et al.	2013	23383113

Al-Minshawy & El-Mazary	2014	24568362
Chen et al.	2014	24670221
Duchatelet et al.	2014	24452206
Duchatelet et al.	2014	24606194
Eberhardt et al.	2014	24311784
Luo et al.	2014	24433512
Tseng et al.	2014	24712020
Meijer et al.;<br>Kim et al.	2014;<br>2015	23893323;<br>26486037
Emery et al.	2015	25995458
Huh et al.	2015	26315201
Namer et al.	2015	25993546
Patel et al.	2015	26528699
Russo et al.	2015	26609309
Cao et al.	2016	27099175
Hoeijmakers et al.	2016	27660061
Huang et al.	2016	27653502
Kundu et al.	2016	27799815
McDonell et al.	2016	26920677
Yang et al.	2016	27413160
Geha et al.;<br>Geha et al.	2016;<br>2018	27088781;<br>31080911
Farrar et al.	2017	28134657
Lee et al.	2017	27699862
Tanaka et al.	2017	28381558
Tham et al.	2017	28751508
Wu et al.	2017	28990532
Kollipara et al.	2018	DOI: 10.4103/JDRNTRUHS.JDRNTRUHS_7_18
Wang et al.	2018	30126782
Willekens et al.	2018	30006649
Arthur et al.	2019	30416015
Cinats & Haber	2019	31259429
Huppke et al.	2019	31132363
Ito et al.	2019	31517003
Lee et al.	2019	30976641
López-Valverde et al.	2019	WOS: 000493180700009
Tjahjono et al.	2019	29524220
Faignart et al.	2020	32723684
Fan et al.	2020	32652278
Greco et al.	2020	31895432
Kharkar et al.	2020	DOI: 10.18203/issn.2455-4529.IntJResDermatol20203758
Shinkarevsky Fleitman et al.	2020	32133669
Lam et al.	2021	34353840
Nwebube et al.	2021	33688580
Sooy et al.	2021	33602765
Assaf & Winters	2022	37483401
Feng et al.	2022	36052366
Li et al.	2022	35282159
Mamatsi et al.	2022	36128208
Matarneh & Witman	2022	34888934
Montoya et al.	2022	35421007
Phong et al.	2022	36406307
Reese et al.	2022	35155035

Tolley & Walsh	2022	36168350
Wang et al.	2022	35461983
Ye et al.	2022	35620189
Bourkas et al.	2023	38022850
Deuis et al.	2023	37721535
El Khatib et al.	2023	36669785
Giaimo et al.	2023	33316170
Loureiro et al.	2023	36950140
Michelerio et al.	2023	37557164
Sun et al.	2023	37628281
Watabe et al.	2023	36815391
Yuan et al.	2023	37555797
Zuo et al.	2023	37273700

Merged references with overlapping case(s) are presented within the same row. PMID, PudMed ID is presented unless specified otherwise. DOI, Digital Object Identifier; N/A, Not Available; NLM, National Library of Medicine; WOS, Web of Science

Supplementary Table 3. Abnormal clinical investigations reported in studies

	Number of studies with reported results (S = 167)	Number of cases with reported results (C = 411)
**Laboratory tests**		
Abnormal vitamin B12 and/or D levels	2 (1.2)	16 (3.9)
Anemia	1 (0.6)	1 (0.2)
Bacterial cultures	2 (1.2)	2 (0.5)
Defect in immunity	5 (3.0)	7 (1.7)
Elevated blood urea	1 (0.6)	1 (0.2)
Elevated liver enzymes	5 (3.0)	5 (1.2)
Elevated urinary hydroxymethyl mandelic acid	1 (0.6)	1 (0.2)
Hematuria	1 (0.6)	1 (0.2)
High cholesterol	2 (1.2)	2 (0.5)
High cerebrospinal fluid glucose	1 (0.6)	1 (0.2)
High lactate	1 (0.6)	1 (0.2)
High minerals/electrolytes	2 (1.2)	2 (0.5)
High platelet count	13 (7.8)	21 (5.1)
High red blood cells	1 (0.6)	1 (0.2)
High white blood cells	6 (3.6)	6 (1.5)
Hypernatremia	1 (0.6)	1 (0.2)
Hypoglycemia	1 (0.6)	1 (0.2)
Increase ereatine phosphokinase	1 (0.6)	1 (0.2)
Increased erythrocyte sedimentation rate / C reactive protein	11 (6.6)	16 (3.9)
Increased hormones	1 (0.6)	1 (0.2)
Increased lead levels	2 (1.2)	2 (0.5)
Increased urinary catecholamine levels	1 (0.6)	1 (0.2)
Low iron	2 (1.2)	2 (0.5)
Low proteins	1 (0.6)	1 (0.2)
Positive antinuclear antibodies	8 (4.8)	16 (3.9)
Positive lupus anticoagulant	1 (0.6)	1 (0.2)
Positive monospot test	1 (0.6)	1 (0.2)
Positive Rheumatoid factor	1 (0.6)	1 (0.2)
Proteinuria	2 (1.2)	2 (0.5)
Reduced growth hormone and insulin-like growth factor-1	1 (0.6)	1 (0.2)
**Neurological examinations**		
Abnormal nerve conduction	9 (5.4)	12 (2.9)
Abnormal quantitative sensory testing	23 (13.8)	49 (11.9)
Hyperexcitability of nerves	2 (1.2)	10 (2.4)
Hyperkeratosis	9 (5.4)	11 (2.7)
Hypertension	8 (4.8)	12 (2.9)
Hypotension	3 (1.8)	3 (0.7)
Inflammation of blood vessels	21 (12.6)	25 (6.1)
Low nerve fiber density	7 (4.2)	13 (3.2)
Restless legs syndrome	1 (0.6)	1 (0.2)
Small fiber neuropathy	9 (5.4)	15 (3.6)
Sudomotor dysfunction	11 (6.6)	24 (5.8)
**Vascular examinations**		
Abnormal morphology	1 (0.6)	1 (0.2)
Increased blood flow	6 (3.6)	10 (2.4)
Increased temperature	14 (8.4)	25 (6.1)
Reduced blood flow	3 (1.8)	4 (1)

Data presented as n (%).

Supplementary Table 4. Pharmacological approaches reported in studies without or unreported beneficial response

	Number of cases with reported treatments (C = 411)	Beneficial treatment response (C=411)	
		No	Not reported
**Pharmacotherapy**			
Unspecified ACE inhibitors	3 (0.7)	1 (33.3)	2 (66.7)
Adrenergic antagonists			
Methyldopa	1 (0.2)	1 (100)	0
Dexmedetomidine	1 (0.2)	0	1 (100)
Ergotamine tartrate	2 (0.5)	2 (100)	0
Guanfacine	1 (0.2)	0	1 (100)
Midodrine	1 (0.2)	1 (100)	0
Noradrenaline	1 (0.2)	1 (100)	0
Adrenergic antagonists			
Doxazosin	4 (1)	2 (50.0)	2 (50.0)
Reserpine	1 (0.2)	1 (100)	0
Unspecified alpha and beta blockers	2 (0.5)	2 (100)	0
Anesthetics			
Tremicaine	1 (0.2)	1 (100)	0
Antibiotics			
Amikacin	1 (0.2)	1 (100)	0
Befuxin	1 (0.2)	1 (100)	0
Ceftriaxone	1 (0.2)	0	1 (100)
Meropenem	1 (0.2)	0	1 (100)
Methicillin	1 (0.2)	0	1 (100)
Metronidazole	1 (0.2)	0	1 (100)
Penicillin	1 (0.2)	1 (100)	0
Piperacillin	2 (0.5)	0	2 (100)
Polymyxin B	1 (0.2)	1 (100)	0
Tazobactam	2 (0.5)	0	2 (100)
Tetracycline	1 (0.2)	1 (100)	0
Trimethoprim	1 (0.2)	0	1 (100)
Vancomycin	3 (0.7)	0	3 (100)
Antidiuretics			
Pitressin	1 (0.2)	1 (100)	0
Antiemetics			
Ondansetron	1 (0.2)	0	1 (100)
Pantoprazole	1 (0.2)	1 (100)	0
Unspecified proton pump inhibitor	1 (0.2)	0	1 (100)
Antifungals			
Fluconazole	1 (0.2)	0	1 (100)
Antihistamines			
Antazoline	1 (0.2)	1 (100)	0
Chlorpheniramine maleate	1 (0.2)	1 (100)	0
Fexofenadine	1 (0.2)	1 (100)	0
Phernergan nocte/ promethazine	1 (0.2)	1 (100)	0
Trimeprazine	1 (0.2)	1 (100)	0
Antivirals			
Acyclovir	1 (0.2)	0	1 (100)
Beta-blockers			
Metoprolol	2 (0.5)	2 (100)	0
Calcium channel blockers			
Diltiazem	1 (0.2)	1 (100)	0

Cannabinoids			
Cannabidiol	1 (0.2)	1 (100)	0
Cholinergic agonists			
Carbachol	1 (0.2)	1 (100)	0
Nicotine	1 (0.2)	1 (100)	0
Cholinergic antagonists			
Atropine	1 (0.2)	1 (100)	0
Corticosteroids			
Budesonide	1 (0.2)	0	1 (100)
Cortisone	1 (0.2)	1 (100)	0
Fludrocortisone	1 (0.2)	0	1 (100)
Triamcinolone	1 (0.2)	1 (100)	0
COX inhibitors			
Clinoril	1 (0.2)	1 (100)	0
Ibuprofen	18 (4.4)	8 (44.4)	10 (55.6)
Ketoprofen	1 (0.2)	0	1 (100)
Metamizole	1 (0.2)	1 (100)	0
Misoprostol	7 (1.7)	5 (71.4)	2 (28.6)
Tolmetin	1 (0.2)	1 (100)	0
Diuretics	1 (0.2)	1 (100)	0
Dopamine and/or serotonin agonists			
Aripiprazole	1 (0.2)	1 (100)	0
Rizatriptan	5 (1.2)	0	5 (100)
Olanzapine	1 (0.2)	1 (100)	0
Emollients			
Ointments	4 (1.0)	4 (100)	0
Topical keratolytics	2 (0.5)	2 (100)	0
GABA reuptake inhibitors			
Tiagabine	2 (0.5)	2 (100)	0
GABA-A receptor agonists			
Alprazolam	1 (0.2)	1 (100)	0
Amytal	1 (0.2)	1 (100)	0
Flunitrazepam	1 (0.2)	1 (100)	0
Phenobarbital	1 (0.2)	1 (100)	0
Propofol	1 (0.2)	1 (100)	0
Immunosuppressants			
Methotrexate	1 (0.2)	0	1 (100)
Mycophenolate	1 (0.2)	0	1 (100)
Rapamycin	1 (0.2)	1 (100)	0
Sirolimus	1 (0.2)	1 (100)	0
Unspecified	1 (0.2)	0	1 (100)
Keratin inhibitors			
Oral retinoids	2 (0.5)	2 (100)	0
Laxatives			
Polyethylene glycol	1 (0.2)	0	1 (100)
Medicinal plants	1 (0.2)	1 (100)	0
Traditional Chinese medicines	2 (0.5)	1 (50.0)	1 (50.0)
NDRIs			
Methylphenidate	2 (0.5)	0	2 (100)
Neurotrophin growth agonists	1 (0.2)	1 (100)	0
Nitric oxide	1 (0.2)	0	1 (100)
Opioid receptor agonists			
Dihydrocodeine	2 (0.5)	2 (100)	0

Hydrocodone	3 (0.7)	1 (33.3)	2 (66.7)
Meperidine	4 (1.0)	1 (25.0)	3 (75.0)
Nalbuphine	1 (0.2)	1 (100)	0
Oxycontin	1 (0.2)	0	1 (100)
Pentazocine	1 (0.2)	0	1 (100)
Piritramide	1 (0.2)	1 (100)	0
Propoxyphene	2 (0.5)	2 (100)	0
Sufentanil	1 (0.2)	1 (100)	0
Tapentadol	1 (0.2)	0	1 (100)
Platelet activation inhibitors			
Pentoxifylline	2 (0.5)	1 (50.0)	1 (50.0)
Prostacyclin	1 (0.2)	1 (100)	0
Sex hormone agonists			
Estrogen	1 (0.2)	1 (100)	0
Diethylstilbestrol	1 (0.2)	1 (100)	0
Sodium channel blockers			
EMLA cream	1 (0.2)	1 (100)	0
Furosemide	1 (0.2)	0	1 (100)
Hydantoin	3 (0.7)	3 (100)	0
Phenytoin	2 (0.5)	1 (50.0)	1 (50.0)
Topiramate	1 (0.2)	0	1 (100)
Zonisamide	1 (0.2)	1 (100)	0
Unspecified	1 (0.2)	1 (100)	0
SSRIs/SNRIs			
Citalopram	1 (0.2)	1 (100)	0
Doxepin	2 (0.5)	2 (100)	0
Fluoxetine	3 (0.7)	1 (33.3)	2 (66.7)
Trazodone	1 (0.2)	1 (100)	0
Supplements			
Alfacalcidol	1 (0.2)	1 (100)	0
Calcium	2 (0.5)	1 (50.0)	1 (50.0)
Cholecalciferol	1 (0.2)	0	1 (100)
L-tryptophan	1 (0.2)	1 (100)	0
Mecobalamin	1 (0.2)	1 (100)	0
Multivitamin	1 (0.2)	0	1 (100)
Thyroid extract	1 (0.2)	1 (100)	0
Thyroxine	1 (0.2)	0	1 (100)
Vitamin B combination therapy	1 (0.2)	0	1 (100)
Vitamin B6	1 (0.2)	0	1 (100)
SV2A protein binding			
Levetiracetam	1 (0.2)	0	1 (100)
TRPM8 receptor agonist			
1% menthol in aqueous cream	1 (0.2)	1 (100)	0
Unspecified Vasoconstrictors	1 (0.2)	1 (100)	0
Vasodilators			
Beraprost	1 (0.2)	1 (100)	0
Buflomedil	1 (0.2)	1 (100)	0
Dipyridamole	3 (0.7)	3 (100)	0
Iloprost	1 (0.2)	1 (100)	0
Minoxidil	1 (0.2)	0	1 (100)
Wound dressings			
Granuflex	1 (0.2)	1 (100)	0
Trimovate cream	1 (0.2)	1 (100)	0

Unspecified wound dressing	1 (0.2)	1 (100)	0

Data presented as n (%).
ACE, Angiotensin-converting enzyme; COX, cyclooxygenase; EGFR, epidermal growth factor receptor; GABA, gamma-aminobutyric acid; NDRI, Norepinephrine and dopamine reuptake inhibitor, SSRI, Selective serotonin reuptake inhibitor; SNRI, Serotonin and norepinephrine reuptake inhibitor; SV2A, Synaptic vesicle glycoprotein 2A; TRPM, transient receptor potential of the melastatin subtype.
